# Predictors of Transplant Regret: A Case–Control Study Nested Within a Prospective Cohort of HSCT Recipients

**DOI:** 10.1002/cam4.70828

**Published:** 2025-04-01

**Authors:** Janae L. Kirsch, James R. Cerhan, William J. Hogan, Holly C. Edwards, Christi A. Patten, Tabetha Brockman, Christine Hughes, Angela Dispenzieri, Stephen M. Ansell, Dennis A. Gastineau, Shawna L. Ehlers

**Affiliations:** ^1^ Department of Psychiatry and Psychology Mayo Clinic Rochester Minnesota USA; ^2^ Division of Epidemiology, Department of Health Sciences Research Mayo Clinic Rochester Minnesota USA; ^3^ Division of Hematology, Department of Medicine, College of Medicine Mayo Clinic Rochester Minnesota USA; ^4^ Behavioral Health Research Program Mayo Clinic Rochester Minnesota USA

**Keywords:** health status, hematopoietic stem cell transplant, illness perception, oncology, transplant regret

## Abstract

**Objective:**

To explore pre–hematopoietic stem cell transplant (HSCT) demographic, disease, and psychological factors predictive of future transplant regret and to determine post‐HSCT variables associated with regret.

**Patients and Methods:**

HSCT candidates participated in a prospective cohort study (June 2008–October 2013) examining health behaviors and HSCT outcomes, including completion of standardized surveys at pre‐HSCT (baseline) and 1‐year post‐HSCT. Cases were participants that endorsed regret at 1‐year post‐HSCT follow‐up, and controls were participants without regret at 1 year, matched on age, sex, and transplant type. For cases and controls, pre‐HSCT psychosocial evaluations were abstracted from the electronic health record and coded to determine the Psychosocial Assessment of Candidates for Transplantation score, psychosocial stressors, and mental health diagnoses. The association of selected factors with regret was estimated with odds ratios and 95% confidence intervals from conditional logistic regression models.

**Results:**

At post‐HSCT, 49 participants of 638 endorsed transplant regret (8%) and formed the case group; 98 controls were matched from remaining participants. Cases and controls were well matched on age (56.6 vs. 57.2 years), sex (both groups 34.7% female), and transplant type (both groups 81.6% autologous). After controlling for the number of hospitalizations and active treatment status, conditional logistic regression revealed that patients who endorsed regret were 3.7 times (95% CI = 1.37–9.69, *p* = 0.008) more likely to not be in remission compared to controls at 1‐year post‐HSCT.

**Conclusion:**

Matched case–control analyses revealed that no pre‐HSCT variables collected during the pre‐HSCT evaluation period were predictive of transplant regret, while poorer outcomes at 1‐year after transplant were associated with regret.

**Abbreviations**
ECOG: Eastern Cooperative Oncology Group Performance Status Scale
FACT‐BMT: Functional Assessment of Cancer Therapy—Bone Marrow Transplant
GVHD: graft‐versus‐host disease
HSCT: hematopoietic stem cell transplant
PACT: Psychosocial Assessment of Candidates for Transplantation

## 1 Introduction

Hematopoietic stem cell transplant (HSCT) is a well‐established and increasingly utilized procedure to address malignant and non‐malignant conditions [1]. Despite being a potentially life‐saving procedure, the intense nature of HSCT‐related procedures challenges patients' ability to adhere to medical interventions and healthcare provider recommendations prior to and well after HSCT [2, 3, 4]. Intensive conditioning regimes, high chemotherapy doses that may cause drug toxicities, infections, and immunodeficiency complications as well as the long recovery process post‐HSCT, remain common experiences for many patients [5, 6].

Depending on the clinical presentation, individuals may have several treatment options to consider, while others may be debating between pursuing HSCT and palliative measures. Given the treatment decisions individuals with cancer must make, regret has been an area of interest for researchers across varying cancer populations [7]. Regret, as it relates to cancer and medical decision making, has been defined several ways; however, Connolly and Reb have noted that most definitions include four parts: (1) regret will be avoided when possible, (2) it includes emotional and cognitive components, (3) it is unique from concepts of negative affect including disappointment, and (4) there is a comparison between the decision and another option [8].

Although much of the regret literature has looked at non‐hematological cancer populations [7], a small body of research has examined regret in patients undergoing HSCT [9, 10]. Overall, these studies suggest that regret occurs in a small percentage of cases. In a study exploring regret at 100 days, 5 months, and 12 months post‐HSCT, 6%–8% of patients endorsed regret at each timepoint [9]. In a sample of HSCT survivors (mean years since transplant = 5.7 years, SD = 3.4, range = 1–14 years), 9.2% of patients who underwent allogenic transplantation reported regret in the past 7 days [10]. Findings suggest that individuals with lower quality of life and household income, greater depressive symptoms and number of medical care visits, and those who experienced graft‐versus‐host disease (GVHD) or recurrence following transplant were more likely to endorse regret [9, 10]. In a qualitative study of 25 patients exploring the impact of experimental stem cell transplant, no patients reported regret related to undergoing transplant because HSCT was viewed as an opportunity to lengthen their lives or as their only option [11]. However, themes of regret did emerge relative to post‐transplant physical and cognitive changes [11]. These findings underscore the complex decision‐making process to pursue HSCT as well as the evolving emotional and cognitive appraisals of the decision following the procedure.

Given the small number of studies that have examined HSCT regret, and the varying methodologies used, the prevalence and predictors of transplant regret are unknown. Therefore, the first aim of the study was to document the prevalence of regret and explore demographic, disease, and psychological factors at pre‐transplant that may be predictive of regret 1‐year post‐HSCT. Our second aim was to determine if post‐transplant variables were associated with regret 1‐year post‐HSCT. Additionally, we sought to address two gaps in the current literature by including individuals who received either allogeneic or autologous transplant and by utilizing multiple sources of information including medical record abstraction, self‐report, and mental health data obtained by diagnostic interview with a licensed mental health provider. We hypothesized that the individuals who had greater psychological burden (e.g., mental health diagnosis), more stressors, lower Psychosocial Assessment of Candidates for Transplantation (PACT) score, and those with greater disease severity at pre‐transplant would be more likely to endorse future regret. We also hypothesized that individuals with complications post‐transplant (e.g., GVHD, unplanned hospitalizations) would be significantly more likely to endorse regret.

## 2 Methods

The parent study was a prospective cohort study of 1000 individuals preparing to undergo HSCT at a large academic medical center in the Midwest that examined health behaviors prior to and 1 year following HSCT. Given the significant resources required to abstract psychological and social work records, we conducted a case–control analysis nested within the cohort. Nested case–control studies are advantageous in situations such as this while minimally impacting statistical performance [12]. Age, sex, and transplant type were selected as matching variables through discussions with our multidisciplinary team with the goal of reducing the chance of biased comparisons in the nested sample. The advantage of matching is to optimize group equivalency when studying a rare outcome. All participants provided written informed consent. The protocol was approved by the Mayo Clinic Institutional Review Board.

Of the 1000 people who enrolled in the parent study, 870 (87%) completed study surveys prior to HSCT (defined as baseline). Among the people who completed baseline questionnaires, 638 (73%) completed and returned the 1‐year follow‐up survey. Forty‐nine of these participants endorsed regret (cases). The 49 cases were matched 1:2 with participants who did not endorse regret at the 1‐year follow‐up [13, 14] which resulted in a final sample size of 147. We successfully matched all participants on age (±2 years), sex, and transplant type (allogeneic vs. autologous) to account for potential confounding variables.

Following matching, psychosocial clinical data, including psychological and social work records in the year prior to transplantation, were reviewed and abstracted. Psychological evaluations included psychiatric diagnostic interviews inclusive of mental health diagnosis using the Diagnostic and Statistical Manual of Mental Disorders IV (DSM‐IV) [15]. Two authors (J.L.K., H.C.E.) independently rated each participant on the PACT [16, 17] measure based on the available clinical records. Rating disagreements were discussed with the senior author (S.L.E.), and consensus was achieved.

### 2.1 Measures

#### 2.1.1 Demographic and Disease Characteristics

Demographic characteristics and hospitalizations were obtained through self‐report, respectively, at baseline and 1‐year post‐HSCT. Hospitalizations were defined as staying at least one night in the hospital. Transplant‐specific information (e.g., conditioning regimen, GVHD, disease) was obtained from the institution's transplant center, which collects data related to the transplant procedure and its outcomes.

#### 2.1.2 Functional Assessment of Cancer Therapy—Bone Marrow Transplant (FACT‐BMT)

The FACT‐BMT assesses health‐related quality of life in transplant recipients [18] and was completed at 1‐year post‐HSCT. The survey assesses several domains including physical, social, emotional, and functional wellbeing as well as provides a transplant‐specific subscale [18]. The FACT‐BMT also assesses factors that are not included in the subscale scores including, “I regret having the bone marrow transplant” which was used to quantify the presence of transplant regret in this sample.

#### 2.1.3 Psychosocial Assessment of Candidates for Transplantation (PACT)

The PACT was originally developed to rate solid organ transplant acceptability [19], but was adapted for use in HSCT populations [16, 17]. It has been recognized as a useful tool for identifying patients who may need support or resources. The measure assesses candidate acceptability on eight domains including family/support system stability and availability, psychopathology, risk for psychopathology, healthy lifestyle, drug and alcohol use, compliance with medication and medical advice, and knowledge and education of the transplant process. Final PACT scores range from 0 (poor candidate) to 4 (excellent candidate).

### 2.2 Statistical Analyses

IBM SPSS for Windows, Version 28 (IBM Corp., Armonk, NY, USA) was used to conduct statistical analyses. Participant demographic, disease, and quality of life characteristics were summarized using descriptive statistics. Responses to several variables (e.g., ECOG, race, PACT score, number of hospitalizations) were collapsed due to small cell counts to dichotomous variables. Conditioning regimen was collapsed into three groups: BEAM, Melphalan, and Other. To explore group differences between those who endorsed regret and those who did not endorse transplant regret, conditional logistic regressions with 95% confidence intervals were performed. A multivariate conditional logistic regression was run to explore the odds of regret for all variables that were significant in univariate models.

## 3 Results

Approximately 8% of the individuals (*n* = 49) in the original cohort who underwent HSCT transplant reported regret at 1 year. Individuals who endorsed transplant regret tended to be middle aged (mean = 57 years ±10 years), White (*n* = 44, 90%), married or partnered (*n* = 44, 90%), diagnosed with multiple myeloma (*n* = 22, 45%) and underwent autologous transplant (*n* = 40, 82%). Forty‐five percent of people who endorsed regret were still completing treatment 1‐year post‐transplant, and 61% had experienced at least one hospitalization.

Cases and controls were well matched on age (56.6 vs. 57.2 years), sex (34.7% female for both groups), and transplant type (81.6% autologous for both groups). Forty percent rated their health at 1‐year post‐HSCT as “excellent” or “very good” and over half of patients reported no hospitalizations in the year since transplant. Disease and demographic data by group are provided in Table 1.

**TABLE 1:** Disease and demographic characteristics.

Characteristic	Regret (cases) (*n* = 49)		No regret (controls) (*n* = 98)	
	*n*	%	*n*	%
Age at transplant,^a^ mean (SD)	56.58 (10.32)		57.19 (10.12)	
Sex^a^				
Male	32	65.3	64	65.3
Female	17	34.7	34	34.7
Race				
White	44	89.8	94	95.9
Black	1	2.0	1	1.0
Asian/South Pacific Islander	1	2.0	0	0.0
Other	1	2.0	1	1.0
Unknown/choose not to disclose	2	4.1	2	2.0
Education				
High school degree/GED or less	14	28.6	26	26.5
Some college	14	28.6	24	24.5
4‐year degree	6	12.2	17	17.3
Post‐graduate studies	12	24.5	28	28.6
Missing	3	6.1	3	3.1
Employment				
Not employed	24	49.0	49	50.0
Employed/self‐employed	25	51.0	45	45.9
Missing	0	0.0	4	4.1
Relationship status				
Married/partnered	44	89.8	82	83.7
Other	5	10.2	11	11.2
Missing	0	0.0	5	5.1
Diagnosis				
Acute leukemia	5	10.2	8	8.2
Amyloidosis	5	10.2	7	7.1
Multiple myeloma	22	44.9	42	42.9
Non‐Hodgkin lymphoma	11	22.4	27	27.6
Other	6	12.2	14	14.3
ECOG				
0	24	49.0	48	49.0
1	24	49.0	42	42.9
2	1	2.0	8	8.2
PACT				
0–1	4	8.1	7	7.1
2	18	36.7	32	32.7
3	20	40.8	38	38.8
4	7	14.3	21	21.4
Presence of 1+ mental health diagnosis	19	38.7	51	41.8
Number of psychosocial stressors				
1	17	34.7	32	32.7
2–4	13	26.2	39	39.8
Conditioning regimen				
BEAM	14	28.6	30	30.6
Carboplatin/etoposide	0	0	1	1.0
Melphalan	27	55.1	57	58.2
Busulfan/cytoxan	1	2.0	3	3.1
Busulfan/fludarabine	1	2.0	0	0.0
Cytoxan/TBI	3	6.1	5	5.1
Cytoxan/TBI/ATG/fludarabine	1	2.0	0	0.0
Fludarabine/TBI	2	4.1	2	2.0
Transplant type^a^				
Autologous	40	81.6	80	81.6
Allogeneic	9	18.4	18	18.4
Post‐HSCT variables				
Acute GVHD	7	14.3	10	10.2
Chronic GVHD	1	2.0	7	7.1
Complete remission^b^	14	28.6	63	64.3
Currently on treatment^b^	22	44.9	20	20.4
Hospitalization in past year^b^	30	61.2	32	32.7
Self‐report health status^b^				
Excellent/very good	6	12.2	53	54.1
Good	17	34.7	24	24.5
Fair/poor	26	53.1	17	17.3

^a^ Denotes matching factors.

^b^ Measurement taken at 1‐year post‐HSCT.

Prior to HSCT, 73% of patients completed a psychological evaluation with a diagnostic interview to detect the presence of mental health diagnoses and other psychosocial stressors that may impact the transplant trajectory. The overall prevalence at the pre‐HSCT evaluation of at least one mental health diagnosis was similar between the regret and no regret groups (39% and 42%, respectively). Although the cell sizes were small, the group of patients who went on to develop regret had a higher prevalence of co‐occurring mental health diagnoses at baseline (16% vs. 8%). The median PACT score for patients who endorsed regret was 3 (mean: 2.59, range: 0–4). For those who did not endorse regret, the median PACT score was also 3 (mean: 2.74, range: 1–4).

Conditional logistic regressions were conducted to examine whether pre‐ and post‐HSCT disease, demographic, and psychosocial variables were associated with post‐HSCT regret at 1‐year follow‐up. There was no association for the number of mental health diagnoses, number of psychosocial stressors (e.g., caregiving/family stress, financial worry, and employment concerns), or PACT score with transplant regret (Table 2). Post hoc analyses did not reveal significant group differences based on neither mental health diagnostic groups (i.e., depressive, anxious, adjustment, and substance use disorders) nor specific psychosocial stressors. Additionally, there were no significant group differences based on disease group, pre‐HSCT ECOG score, or conditioning regimen (Table 2).

**TABLE 2:** Univariate conditional logistic regressions of patients who do and do not endorse transplant regret.

Characteristic	Odds ratio (95% CI)	*p*
Pre‐HSCT variables		
Diagnosis		
Acute leukemia	1.00 (reference)	
Amyloidosis	1.04 (0.06–17.21)	0.98
Multiple myeloma	0.74 (0.05–10.41)	0.82
Non‐Hodgkin lymphoma	0.60 (0.04–8.08)	0.70
Other	0.68 (0.15–3.09)	0.62
ECOG		
0	1.00 (reference)	
1	1.22 (0.56–2.64)	0.62
2	0.21 (0.02–1.90)	0.16
Conditioning regimen		
BEAM	1.00 (reference)	
Melphalan	1.03 (0.48–2.19)	0.94
Other	2.65 (0.48–14.58)	0.26
PACT		
0–1	1.00 (reference)	
2	1.01 (0.25–4.05)	0.99
3	0.95 (0.25–3.66)	0.94
4	0.59 (0.13–2.62)	0.49
Presence of 1+ mental health diagnosis	0.79 (0.30–2.05)	0.63
Number of psychosocial stressors		
1	1.00 (reference)	
2–4	0.60 (0.22–1.63)	0.32
Post‐HSCT variables		
Acute GVHD	2.50 (0.48–13.98)	0.30
Chronic GVHD	0.25 (0.03–2.19)	0.21
Complete remission^a^	5.76 (2.33–14.22)	< 0.001
Currently on treatment^a^	3.00 (1.42–6.34)	0.004
Hospitalization in past year^a^	3.72 (1.69–8.19)	< 0.001

^a^ Measurement taken at 1‐year post‐HSCT.

In contrast, post‐HSCT factors showed a significant pattern of association with transplant regret. Participants endorsing regret 1‐year post‐HSCT were 3.7 times more likely to have been hospitalized at least once in the year following HSCT compared to individuals who did not endorse regret (Table 2). Similarly, patients endorsing regret were 5.8 times more likely to not be in complete remission compared to controls. Patients endorsing regret were three times more likely to report current active treatment compared to controls (who did not endorse regret) (Table 2). There were no significant group differences based on the occurrence of acute or chronic GVHD.

Multivariate conditional logistic regression analyses found that only remission status 1‐year post transplant remained significant in the model (Table 3). After controlling for the number of hospitalizations and active treatment status, patients who endorsed transplant regret were 3.7 times (95% CI = 1.37–9.69, *p* = 0.008) more likely not to be in remission compared to controls at 1‐year post‐HSCT.

**TABLE 3:** Multivariate conditional logistic regression of patients who do and do not endorse transplant regret.

Characteristic	Odds ratio (95% CI)	*p*
Complete remission	3.68 (1.40–9.69)	0.008
Currently on treatment	1.86 (0.80–4.31)	0.151
Hospitalization in past year	2.26 (0.93–5.50)	0.073

Post hoc analyses were conducted to determine if there were any differences in findings based on allogeneic or autologous transplant. Given the small sample size for allogeneic transplant recipients, only univariate models were performed to reduce the risk of overfitting the model. There were no significant findings for any of the variables of interest, which suggests the model was underpowered. For the autologous patients, the same pattern of results was observed in multivariate analyses as was seen in the combined autologous and allogeneic model. Patients who endorsed regret were 3.9 times (95% CI = 1.37–11.30, *p* = 0.011) more likely to not be in remission compared to controls at 1‐year post‐HSCT. Cross‐sectional post hoc analyses were conducted separately from prospective analyses to explore whether regret was associated with emotional wellbeing at the shared timepoint of 1‐year post‐transplant. Regret was associated with lower emotional wellbeing (odds ratio: 0.79, 95% CI = 0.71–0.88, *p* < 0.001). Given that we were interested in examining variables that may be predictive of regret, cross‐sectional variables were not entered into the prospective multivariate analyses.

## 4 Discussion

Approximately 8% of patients in the current study endorsed transplant regret 1‐year post‐HSCT, which is consistent with past research [9, 10]. The prevalence of mental health diagnoses prior to HSCT in cases (39%) and controls (42%) was slightly higher than past research, which found that 32.8% of people in oncology and hematology settings experienced comorbid psychological distress and/or disorder (e.g., depression, anxiety), with the specific rates varying by assessment timing and methodology [20]. Contrary to hypothesis, our nested case–control analysis revealed that mental health diagnosis and pre‐transplant stressors were not significantly associated with regret 1‐year after HSCT. This finding is surprising, as psychological distress has been linked to negative outcomes including higher risk of acute GVHD, greater transplant‐related symptoms, avoidant coping, and shorter survival [21, 22, 23, 24, 25, 26, 27].

However, as expected, standardized measures indicating a lack of improvement in health status after HSCT (i.e., at least one hospitalization, the absence of complete remission) were consistently associated with regret. Concurrent treatment at 1‐year post‐HSCT was also significantly associated with regret, whereas the presence of GVHD was not significant in the models. However, the non‐significant GVHD finding may be due to the small percentage of patients who experienced GVHD in the sample. Overall, these findings suggest that the post‐HSCT clinical course may be the most impactful factor contributing to patients' positive or negative feelings regarding their choice to undergo transplant. It can be theorized that the disappointment or negative emotions associated with a more challenging post‐HSCT clinical course could lead those who are experiencing regret to question whether they had made the correct choice to undergo transplant.

In this sample, regret was most strongly influenced by remission status at 1‐year post‐HSCT. In the multivariate model, only remission status remained significant, with the odds of endorsing regret being almost four times greater for individuals who were not in remission. This is consistent with past research which found that post‐transplant complications and disease relapse were significant predictors of regret [9, 10].

### 4.1 Strengths

Study strengths include a large, prospective cohort of individuals who underwent HSCT, which enabled assessment of regret prevalence as well as embedding a nested case–control study design to better understand factors associated with regret. We incorporated both autologous and allogenic transplant recipients in contrast to past studies of regret in HSCT samples that focused exclusively on allogeneic populations [9, 10]. We quantified mental health diagnoses and psychosocial stressors at pre‐HSCT via abstraction of psychological consult and social work notes in the year leading up to transplant.

### 4.2 Limitations

A major study limitation is the small cell sizes for some comparisons. The coding of PACT score was based on available psychosocial notes in the medical record, rather than quantification of the score in real time by the mental health provider. Although scoring was done by two licensed mental health providers and consensus was achieved, retrospective review of these notes may have led to documentation bias in scoring. Additionally, post‐transplant psychosocial functioning was not assessed. The emotional wellbeing subscale and the transplant regret item both came from the same FACT‐BMT quality of life measure, and as expected, were highly correlated to each other as quality of life domains. Therefore, another limitation of the study was that post‐transplant mood could not be assessed in the model. Transplant regret was quantified by a single item (FACT‐BMT), therefore a more refined analysis of the construct of regret could not be conducted.

### 4.3 Conclusions

The rate of transplant regret in a heterogeneous sample of HSCT recipients at 1‐year follow‐up is consistent with previously published rates in allogeneic populations. In this Midwest sample, transplant regret may be most reflective of the post‐transplant clinical course and perceived health status rather than pre‐transplant psychological and social factors. Given these findings, transplant teams can remain confident that required pre‐transplant evaluation processes appear to be optimized in terms of minimizing future transplant regret. Importantly, the current findings suggest that clinical interventions should focus on post‐transplant screening, especially during key timepoints such as when learning that the disease is not in complete remission, when patients are preparing to start another treatment, or during a hospitalization. Based on the current knowledge, the best way to decrease regret is to maximize HSCT success. However, additional research is needed to better operationalize transplant regret and enhance understanding of the transplant regret trajectory, including how pre‐ and post‐HSCT factors may contribute to feelings of regret across the cancer care continuum.

## Author Contributions

**Janae L. Kirsch:** conceptualization (equal), formal analysis (equal), methodology (equal), writing – original draft (equal), writing – review and editing (equal). **James R. Cerhan:** conceptualization (equal), methodology (equal), supervision (equal), writing – review and editing (equal). **William J. Hogan:** writing – review and editing (supporting). **Holly C. Edwards:** data curation (supporting), writing – review and editing (supporting). **Christi A. Patten:** resources (equal), writing – review and editing (equal). **Tabetha Brockman:** data curation (supporting), writing – review and editing (supporting). **Christine Hughes:** data curation (supporting), writing – review and editing (equal). **Angela Dispenzieri:** writing – review and editing (supporting). **Stephen M. Ansell:** writing – review and editing (supporting). **Dennis A. Gastineau:** data curation (equal), writing – review and editing (supporting). **Shawna L. Ehlers:** conceptualization (equal), funding acquisition (lead), investigation (lead), methodology (equal), project administration (lead), supervision (equal), writing – review and editing (equal).

## Ethics Statement

The protocol was approved by the Mayo Clinic Institutional Review Board.

## Consent

All participants provided written informed consent.

## Conflicts of Interest

The authors declare no conflicts of interest.

## Data Availability

The data that support the findings of this study are available from the corresponding author, upon reasonable request.

## References

[1] A. D'Souza , C. Fretham , S. J. Lee , et al., “Current Use of and Trends in Hematopoietic Cell Transplantation in the United States,” Biology of Blood and Marrow Transplantation 26, no. 8 (2020): e177–e182. 32438042 10.1016/j.bbmt.2020.04.013 PMC7404814

[2] B. Gresch , M. Kirsch , K. Fierz , et al., “Medication Nonadherence to Immunosuppressants After Adult Allogeneic Haematopoietic Stem Cell Transplantation: A Multicentre Cross‐Sectional Study,” Bone Marrow Transplantation 52, no. 2 (2017): 304–306. 27841860 10.1038/bmt.2016.262

[3] L. L. Ice , G. T. Bartoo , K. B. McCullough , et al., “A Prospective Survey of Outpatient Medication Adherence in Adult Allogeneic Hematopoietic Stem Cell Transplantation Patients,” Biology of Blood and Marrow Transplantation 26, no. 9 (2020): 1627–1634, 10.1016/j.bbmt.2020.05.020. 32505809

[4] N. Khera , E. J. Chow , W. M. Leisenring , et al., “Factors Associated With Adherence to Preventive Care Practices Among Hematopoietic Cell Transplantation Survivors,” Biology of Blood and Marrow Transplantation 17, no. 7 (2011): 995–1003. 21145404 10.1016/j.bbmt.2010.10.023 PMC3062948

[5] E. A. Copelan , “Hematopoietic Stem‐Cell Transplantation,” New England Journal of Medicine 354, no. 17 (2006): 1813–1826. 16641398 10.1056/NEJMra052638

[6] E. Hatzimichael and M. Tuthill , “Hematopoietic Stem Cell Transplantation,” Stem Cells and Cloning: Advances and Applications 3 (2010): 105. 24198516 10.2147/SCCAA.S6815 PMC3781735

[7] A. K. Szproch and R. Maguire , “A Systematic Review of the Factors Associated With Regret Post‐Cancer Treatment,” Journal of Psychosocial Oncology 40, no. 1 (2022): 1–25, 10.1080/07347332.2020.1844846. 33191874

[8] T. Connolly and J. Reb , “Regret in Cancer‐Related Decisions,” Health Psychology 24, no. 4S (2005): S29–S34, 10.1037/0278-6133.24.4.S29. 16045415

[9] R. N. Cusatis , H. R. Tecca , A. D'Souza , B. E. Shaw , and K. E. Flynn , “Prevalence of Decisional Regret Among Patients Who Underwent Allogeneic Hematopoietic Stem Cell Transplantation and Associations With Quality of Life and Clinical Outcomes,” Cancer 126, no. 11 (2020): 2679–2686, 10.1002/cncr.32808. 32154926 PMC7220834

[10] G. McErlean , C. Tapp , L. Brice , et al., “Decisional Regret in Long‐Term Australian Allogeneic Hematopoietic Stem Cell Transplantation Survivors: A Cross‐Sectional Survey,” Clinical Nursing Research 32, no. 8 (2023): 10547738231180337, 10.1177/10547738231180337. PMC10504814 37329124

[11] D. J. Drevdahl and K. S. Dorcy , “Transitions, Decisions, and Regret: Order in Chaos After a Cancer Diagnosis,” Advances in Nursing Science 35, no. 3 (2012): 222–235, 10.1097/ANS.0b013e318261a7a7. 22722390

[12] V. L. Ernster , “Nested Case‐Control Studies,” Preventive Medicine 23, no. 5 (1994): 587–590, 10.1006/pmed.1994.1093. 7845919

[13] D. J. Niven , L. R. Berthiaume , G. H. Fick , and K. B. Laupland , “Matched Case‐Control Studies: A Review of Reported Statistical Methodology,” Clinical Epidemiology 4 (2012): 99–110. 22570570 10.2147/CLEP.S30816 PMC3346204

[14] N. Pearce , “Analysis of Matched Case‐Control Studies,” BMJ 352 (2016): i969, 10.1136/bmj.i969. 26916049 PMC4770817

[15] American Psychiatric Association , Diagnostic and Statistical Manual of Mental Disorders: DSM‐IV‐TR (American Psychiatric Association, 2000).

[16] L. Foster , L. McLellan , L. Rybicki , J. Dabney , M. Visnosky , and B. Bolwell , “Utility of the Psychosocial Assessment of Candidates for Transplantation (PACT) Scale in Allogeneic BMT,” Bone Marrow Transplantation 44, no. 6 (2009): 375–380, 10.1038/bmt.2009.37. 19290003

[17] S. Hong , L. Rybicki , D. Corrigan , et al., “Psychosocial Assessment of Candidates for Transplant (PACT) as a Tool for Psychological and Social Evaluation of Allogeneic Hematopoietic Cell Transplantation Recipients,” Bone Marrow Transplantation 54, no. 9 (2019): 1443–1452. 30696998 10.1038/s41409-019-0455-y PMC6663643

[18] R. McQuellon , G. Russell , D. Cella , et al., “Quality of Life Measurement in Bone Marrow Transplantation: Development of the Functional Assessment of Cancer Therapy‐Bone Marrow Transplant (FACT‐BMT) Scale,” Bone Marrow Transplantation 19, no. 4 (1997): 357–368. 9051246 10.1038/sj.bmt.1700672

[19] M. E. Olbrisch , J. L. Levenson , and R. Hamer , “The PACT: A Rating Scale for the Study of Clinical Decision‐Making in Psychosocial Screening of Organ Transplant Candidates,” Clinical Transplantation 3 (1989): 164–169.

[20] A. J. Mitchell , M. Chan , H. Bhatti , et al., “Prevalence of Depression, Anxiety, and Adjustment Disorder in Oncological, Haematological, and Palliative‐Care Settings: A Meta‐Analysis of 94 Interview‐Based Studies,” Lancet Oncology 12, no. 2 (2011): 160–174. 21251875 10.1016/S1470-2045(11)70002-X

[21] N. Grulke , W. Larbig , H. Kächele , and H. Bailer , “Pre‐Transplant Depression as Risk Factor for Survival of Patients Undergoing Allogeneic Haematopoietic Stem Cell Transplantation,” Psycho‐Oncology 17, no. 5 (2008): 480–487, 10.1002/pon.1261. 17879971

[22] A. El‐Jawahri , Y. B. Chen , R. Brazauskas , et al., “Impact of Pre‐Transplant Depression on Outcomes of Allogeneic and Autologous Hematopoietic Stem Cell Transplantation,” Cancer 123, no. 10 (2017): 1828–1838, 10.1002/cncr.30546. 28102896 PMC5419891

[23] A. El‐Jawahri , J. Pidala , N. Khera , et al., “Impact of Psychological Distress on Quality of Life, Functional Status, and Survival in Patients With Chronic Graft‐Versus‐Host Disease,” Biology of Blood and Marrow Transplantation 24, no. 11 (2018): 2285–2292. 30031937 10.1016/j.bbmt.2018.07.020

[24] A. Barata , B. D. Gonzalez , S. K. Sutton , et al., “Coping Strategies Modify Risk of Depression Associated With Hematopoietic Cell Transplant Symptomatology,” Journal of Health Psychology 23, no. 8 (2018): 1028–1037, 10.1177/1359105316642004. 27106092 PMC5509511

[25] F. Hoodin , J. P. Uberti , T. J. Lynch , P. Steele , and V. Ratanatharathorn , “Do Negative or Positive Emotions Differentially Impact Mortality After Adult Stem Cell Transplant?,” Bone Marrow Transplantation 38, no. 4 (2006): 255–264, 10.1038/sj.bmt.1705419. 16785869

[26] J. L. Kirsch and S. L. Ehlers , “Factor Analysis of the Beck Depression Inventory‐II and Long‐Term Hematopoietic Stem Cell Transplantation Survival Using the Research Domain Criteria Framework,” Transplantation and Cellular Therapy 29, no. 3 (2023): 205, 10.1016/j.jtct.2022.12.012. 36563787

[27] E. S. Costanzo , M. B. Juckett , and C. L. Coe , “Biobehavioral Influences on Recovery Following Hematopoietic Stem Cell Transplantation,” Brain, Behavior, and Immunity 30, no. Suppl (2013): S68–S74, 10.1016/j.bbi.2012.07.005. 22820408 PMC3493826

